# Effect of fatty acids on intracellular pneumocandin B_0_ storage in the fermentation of *Glarea lozoyensis*

**DOI:** 10.1186/s40643-023-00677-w

**Published:** 2023-09-19

**Authors:** Weiting Zhang, Ping Yi, Ying Zhou, Kai Yuan, Xiaojun Ji, Ping Song

**Affiliations:** 1 School of Food Science and Pharmaceutical Engineering, Nanjing Normal University, 2 Xuelin Road, Qixia District, Nanjing, 210034 Jiangsu China; 2 State Key Laboratory of Materials-Oriented Chemical Engineering, College of Biotechnology and Pharmaceutical Engineering, Nanjing University of Technology, No. 5 Xinmofan Rd., Nanjing, 210009 China; 3 Nutrition & Health Research Institute, COFCO Corporation, Beijing, 102209 China

**Keywords:** Transcriptome, Pneumocandin B_0_, Stearic acid, Acetic acid, Lipids, *Glarea lozoyensis*

## Abstract

The natural product pneumocandin B_0_ is the precursor of the antifungal drug caspofungin. To explore the relationship between pneumocandin B_0_ and oil. We found that the addition of 1 g/L of oil to the fermentation medium is more conducive to the production of pneumocandin B_0_. The metabolic reaction mechanism was explored using different fatty acids and the results showed that stearic acid and acetic acid increased the total production of pneumocandin B_0_ by 22.98% and 9.08%, respectively, as well as increasing the content of intracellular lipid droplets. We also analyzed gene expression and pathway differences between the two different fatty acids using transcriptome analyses. The addition of both acetic acid and stearic acid promoted an active pentose phosphate pathway, providing cells with higher intracellular reducing power. We found that the addition of fatty acids can lead to lipid accumulation, and lipid droplets can sequester lipophilic secondary metabolites such as pneumocandin B_0_ to reduce cell damage. These results provide novel insights into the relationship between pneumocandin B_0_ biosynthesis and fatty acids in *G. lozoyensis*. In addition, this study provides important genetic information for improving the yield of pneumocandin B_0_ through a strategy of metabolic engineering in the future.

**Graphical Abstract:**

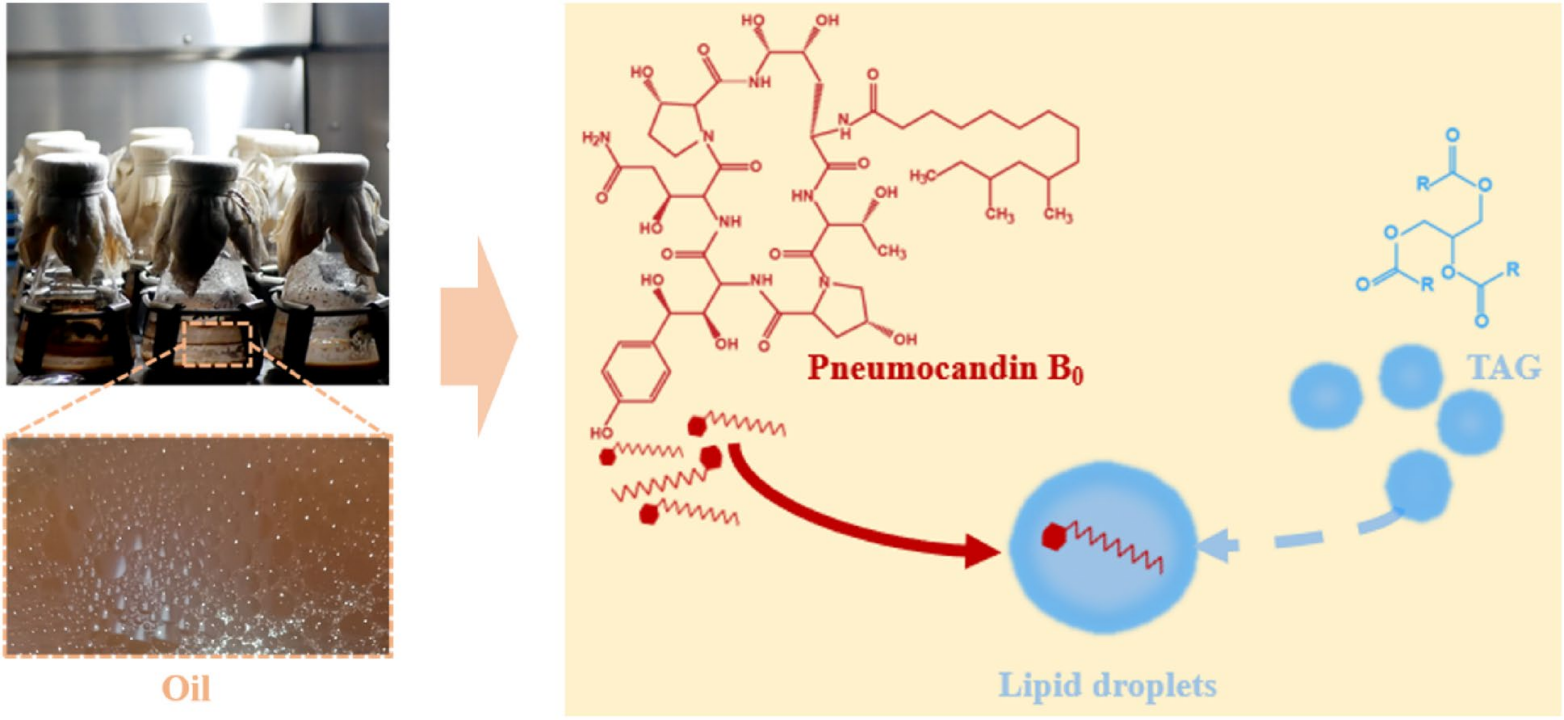

## Introduction

Pneumocandin B_0_, which is produced by the filamentous fungus *Glarea lozoyensis* (Denning 2003), is a precursor of the clinically utilized antifungal drug caspofungin. Caspofungin kills fungi by inhibiting the activity of β-1,3-D-glucan synthetase required for the synthesis of fungal cell walls, disrupting the intracellular and extracellular homeostasis of fungi (Emri et al. 2013). Because its target is unique to the synthesis of fungal cell walls, caspofungin has a good safety profile in humans (Szymanski et al. 2022). Strategies such as mutagenesis (Dong et al. 2021), addition of amino acids (Petersen et al. 2001) or trace elements (Tkacz et al. 1993), and osmotic pressure (Song et al. 2018a, b) control have been used to increase the fermentative yield of pneumocandin B_0_, but the results still lag behind the needs of industrial development.

The low pneumocandin B_0_ yield is attributed to feedback inhibition by the accumulated product, resulting in low titers, cytotoxicity, growth inhibition, and even cell death (Scholler et al. 1993; Hong and Hong 1999). Extractive fermentation is an effective way to relieve intracellular feedback inhibition and reduce cytotoxicity. For example, the addition of SDS as an extractant resulted in a pneumocandin B_0_ yield of 2528.67 mg/L (Yuan et al. 2019). Extractive fermentation in which the extracellular secretion of the product is promoted by the addition of an extractant is referred to as “extracellular extractive fermentation”. To avoid the side effects of their own accumulated secondary metabolites, microorganisms use certain intracellular organelles as ‘reservoirs’, enriching secondary metabolites and thus isolating them from the rest of the cell (Xu et al. 2014). Lipid bodies and membranes are a common intracellular ‘reservoir’ for hydrophobic natural products, such as polyketides and carotenoids (Tan and Liu 2017; Meadows et al. 2016), while their targeted increase is used for “intracellular extraction fermentation”. Oils have great application potential for increasing lipid reservoirs. In general, fatty acids in vegetable oils can be used building blocks for cell membranes (Zang et al. 2011), but they can also provide energy to organisms through β-oxidation (Brzezinska et al. 2022), and they can influence fungal metabolism (Prajapati et al. 2022). Key genes related to fatty acid synthesis and triacylglycerol synthesis overexpressed while also increasing intracellular lipid droplet accumulation, which resulted in a lycopene titer of 70.5 mg per gram of biomass, an increase of 25% over the original strain (Ma et al. 2019). The addition of vegetable oil to the liquid fermentation of the medicinal fungus *Antrodia cinnamomea* could quadruple the yield of triterpenoids (Meng et al. 2021). Lipid droplet biogenesis was promoted and long-chain alkene accumulation was enhanced by overexpressing key genes in the MVA pathway (Ul Hassan et al. 2020). However, no studies tried to improve pneumocandin B_0_ production by adding edible oils to the *G. lozoyensis* fermentation process.

In this study, oil was added to the *G. lozoyensis* fermentation broth, and the effects of adding fatty acids of different chain-length on pneumocandin B_0_ yield and lipid droplet content were investigated. In addition, the differences of transcriptomic expression patterns of cells grown in culture media with the addition of fatty acids with different chain-length were investigated to provide a basis for investigating the underlying molecular mechanisms.

## Materials and methods

### Fungal strain and culture conditions

*G. lozoyensis* CCTCC M 2019020 Q1, stored in the China Center for Type Culture Collection Center, is a mutant derivative of *G. lozoyensis* ATCC 74030. The seed culture medium and conditions were the same as in our previous study (Yuan et al. 2019). The strain was grown on potato glucose agar (PDA) medium at 25 °C. The resulting mycelium was transferred to a 250 mL flask with 45 mL of seed medium, and incubated at 25 °C for 17 days. During flask fermentation, 10% v/v of seed culture was transferred to a 250 mL flask, and 45 mL of fermentation medium was added. The control group did not contain any exogenous fatty acids, and the experimental group was divided into oil and fatty acid addition groups. All oils were purchased from Yihai Kerry Arawana Holdings Co., Ltd., including soybean oil, mixed oil (rapeseed oil, soybean oil), olive oil, sesame oil, and corn oil. The oils and fatty acids were added on the first day of fermentation to a final concentration of 1.0 g/L and the pH was adjusted to 6.8 (Song et al. 2018a, b).

### Biomass, mannitol and pneumocandin B_0_ analysis methods

Samples comprising 5 mL of the culture broth were centrifuged for 10 min at 8000 *g*. The centrifugal pellet was dried at 60 °C for 72 h to a constant weight for biomass calculation (dry cell weight, DCW). The supernatant was used for glucose and mannitol determination on a Shimadzu HPLC system (LC-20AB, CTO-20A, and RID-10A; Shimadzu Corporation, Japan) on an Aminex HPX-87P column (300 mm × 7.8 mm, Bio-Rad, USA), with deionized water as mobile phase at a flow rate of 1 mL/min.

The pneumocandin B_0_ titer was estimated by HPLC. An aliquot comprising 1 mL of the cell-containing fermentation broth was extracted with 4 mL of ethyl alcohol at room temperature on an electronic oscillator for 10 min. The extract was then centrifuged at 8000*g* and 25 °C for 5 min, and the supernatant was analyzed on a Dionex HPLC system (Dionex P680 pump, Chromeleon controller, Dionex UVD 170U Detector; Dionex Corporation, CA, USA). The chromatographic conditions were as follows: ODS column (Venusil MP C18, 4.6 mm × 250 mm, Agela Corporation, Tianjin, China), detection wavelength at 210 nm, at flow rate of 1.0 mL/min, with 0.3%(v/v) phosphoric acid solution (A) and acetonitrile (B) as mobile phase (50% A at 0–10 min, linear gradient from 50% to 25% A in 10 min and 25–5% A in 5 min, keeping 5% A at 15–20 min, 50% A at 20 min keeping at 20–25 min to equilibrate the column). Samples were filtered through a 0.22-µm pore-size single-use membrane prior to injection into the HPLC system (Song et al. 2018a, b).

### Total lipid analysis method

The fatty acid composition was assayed according to the method described in our laboratory earlier (Yuan et al. 2019). A sample comprising 35 mL of the fermentation broth was centrifuged at 4 °C and 8000*g* for 10 min and the supernatant was discarded. The mycelium was washed once with sterile water and then once with the filtrate and with n-hexane spray (to remove residual oil attached to the surface of the organism), and resuspended to the original volume in 50 mL centrifuge tubes. The washed cells were lysed by an ultrasonication, after which 70 mL of freshly prepared extraction reagent (ethanol/hexane, 1:1 v/v) was added to each tube and shaken for 10 min with a vortex shaker. The mixture was centrifuged at 25 °C and 5000*g* for 2 min of to collect the upper hexane–lipid phase, which was concentrated in a rotary evaporator at 65 °C. The obtained lipids were resuspended in 3 mL of saponification solution (0.5 mol/L KOH in methanol). The EP tube was sealed and held at 65 °C for 15 min, after which the mixture was cooled to room temperature. Then, 2 mL of the methylation solution (BF 3-ethyl ether/methanol, 3:7 v/v) was added to the mixture, the EP tube as sealed and incubated at 65 °C for 15 min. At the end of the period, the EP tube was cooled to room temperature, followed by the addition of 2 mL of saturated NaCl solution and 3 mL of hexane. The tubes were mixed by shaking, centrifuged, and the supernatant collected over a membrane, sealed and stored for gas chromatography analysis.

### Extraction of total intracellular RNA

The culture in fermentation medium was incubated until day 10, and samples were taken from each of the three groups, centrifuged, the supernatant discarded, and the pellet stored at − 80 °C. The samples were extracted according to the instructions of TRIzol Reagent, after which the RNA concentration and purity were determined using a Nano Drop spectrophotometer, the RNA integrity was determined by agarose gel electrophoresis, and the RIN value was determined using an Agilent 2100 instrument. The total amount of RNA in a single reaction was 1 µg, with a concentration ≥ 50 ng/μL, and A_260_/A_280_ ratio of 1.8–2.2. The mRNA was treated with Fragmentation buffer and cDNA was synthesized using reverse transcriptase. Then, 2.2% agarose gel was used to recover the appropriately sized fragments for PCR amplification and cDNA libraries were constructed. The initial samples were sequenced on an Illumina HiSeqTM 2000 platform, after which quality control analysis was performed, and clean reads were obtained by filtering. Three parallel tests were performed for all samples.

### Quality control of sequencing data

Sequenced image signals were converted to text files using the Illumina platform and stored as raw data in fastq format. The quality of the raw data was evaluated using SeqPrep and Sickle software, including deletion of the header sequence (5ʹ: AGATGAAGCACGTC; read 3ʹ: AGATGCACGGT), deletion of the uninterpolated reads, and deletion of low-quality bases from the end of the sequence.

### Sequence alignment

The quality control data were compared to the reference genome to obtain mapped data (reads) for subsequent transcript assembly, expression calculation, etc. The RNA sequencing data were first matched to the genome using Bowtie 2, and the results of these matches were used to detect events, such as variable shear and gene fusion. Subsequently, the remaining unmapped reads were then mapped to shear loci based on the local index. Efficient alignment of RNA-Seq reads, especially those spanning multiple exons, was achieved.

### Transcript assembly

Mapped Reads were spliced using Cufflinks software and compared with the original genome annotation information. First, we used the results of the comparison with the reference genome to assemble the sequence, identify PE fragments from different sheaves, find overlapping fragments, build different pathways according to each fragment (containing a point and an edge), and then assemble different transcripts, compare them with the reference transcripts, and obtain information about the differences between this sequencing results and the original annotation.

### Functional annotation of the transcriptomic data

Genes and transcripts were annotated using six major databases: NR (ftp://ftp.ncbi.nlm.nih.gov/blast/db/), SwissProt (http://web.expasy.org/docs/swiss-prot_guideline.html), Pfam (http://pfam.xfam.org/), EggNOG (http://eggnogdb.embl.de/#/app/home), GO (http://www.geneontology.org) and KEGG (http://www.genome.jp/kegg/). DIAMOND software was used to match sequences to genes and transcripts in the NR, SwissProt, and EggNOG databases; BLAST2 GO was used to match sequences to the GO database; HMMER software was used to match sequences to the Pfam database; and KOBAS2.1 was used to obtain KEGG Orthology results.

## Results

### Effects of different oil additives on the production of pneumocandin B_0_ during fermentation

In this study, we tested the addition of five commercially available edible oils, including soybean oil, mixed oil (rapeseed oil, soybean oil), olive oil, sesame oil, and corn oil. At the early stage of fermentation (day 1), 1.0 g/L of oil was added to the culture to investigate the effect of oil on the synthesis of pneumocandin B_0_ and the growth of the strain.

**Fig. 1:**
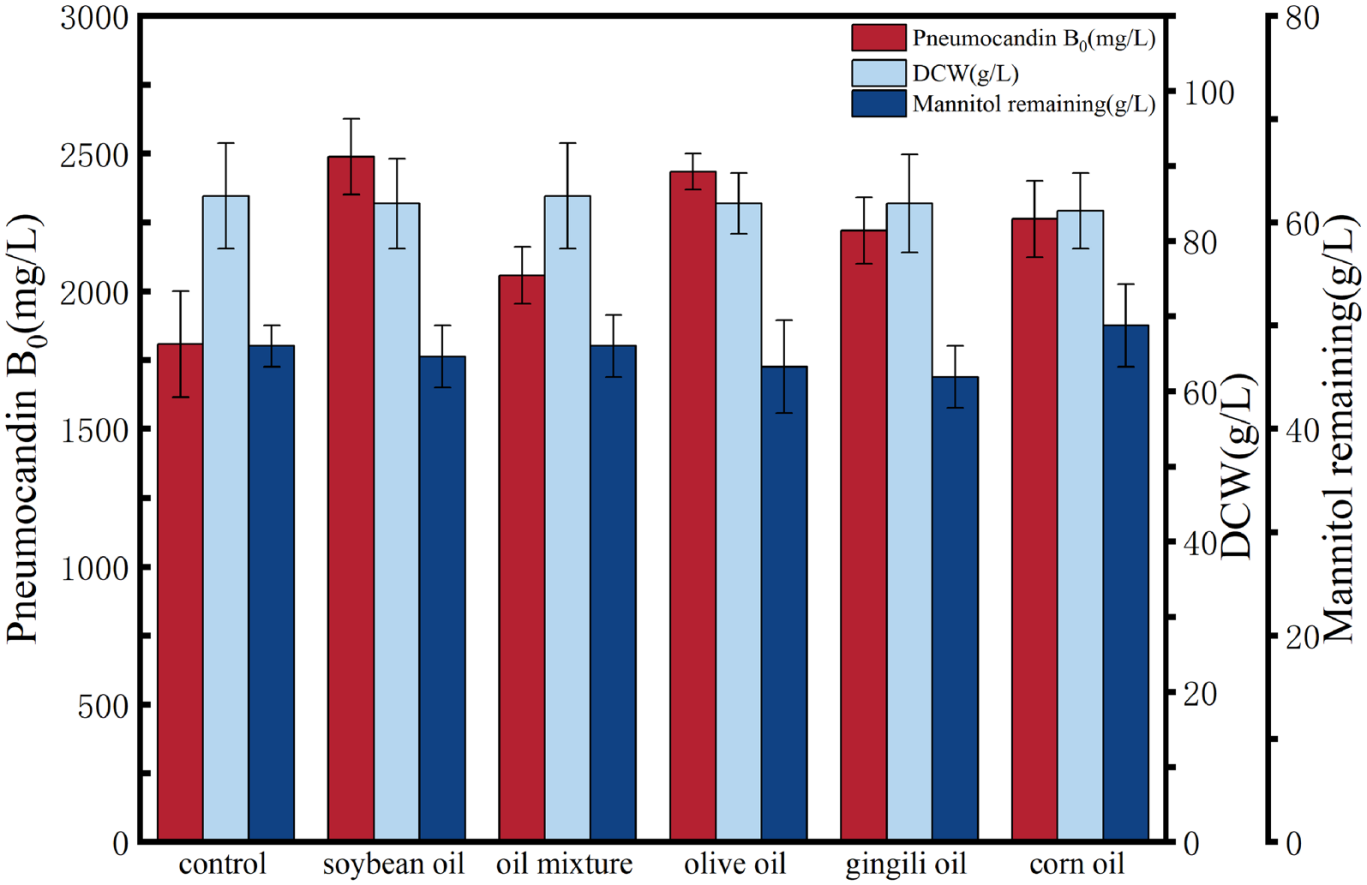
Effects of different lipids on the fermentation of pneumocandin B_0_

The oil addition test was completed after 17 days of fermentation. As shown in Fig. 1, the addition of oil has a significant impact on both pneumocandin B_0_ accumulation and strain growth, but the impact of different oils on the yield of pneumocandin B_0_ varied greatly. The yield from high to low was in the order soybean oil (2487.42 mg/L) > olive oil (2434.29 mg/L) > corn oil (2261.81 mg/L) > sesame oil (2219.41 mg/L) > mixed oil (2056.82 mg/L) > control group (1807.33 mg/L). This is perhaps unsurprising, as the lipid and fatty acid composition of the tested oils also varies greatly.

### Effects of different fatty acids on the yield of pneumocandin B_0_ during fermentation

**Fig. 2:**
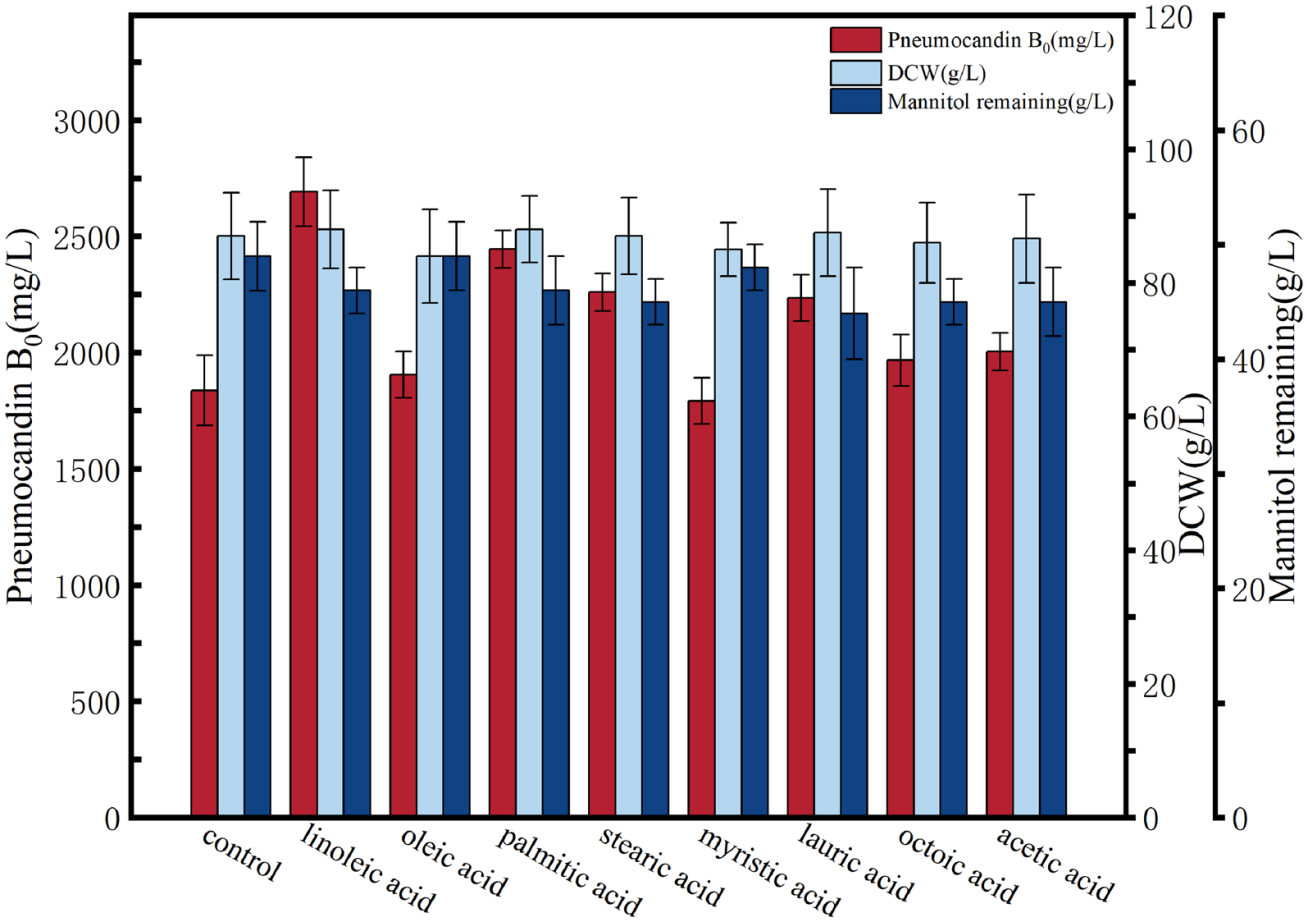
Effects of different fatty acids on the fermentation of pneumocandin B_0_

To explore the specific fatty acids in soybean oil that have the greatest impact on the yield of pneumocandin B_0_, the main components of soybean oil, including linoleic acid, oleic acid, palmitic acid, and stearic acid, were selected. Considering the impact of the carbon chain length of fatty acids on yield, myristic acid, lauric acid, octanoic acid, and acetic acid were also tested as exogenous additives. At the early stage of fermentation (day 1), 1.0 g/L of fatty acids were added to the culture medium to investigate the effects of these different fatty acids on the synthesis of pneumocandin B_0_ and the growth of the fermentation strain. As shown in Fig. 2, the addition of fatty acids with different chain lengths and saturations had similar effects on biomass and residual mannitol as was observed in the oil addition group, without significant differential effects. However, fatty acids with different chain lengths and saturations had a significant impact on the synthesis of pneumocandin B_0_. The yield from high to low was in the order linoleic acid (2691.56 mg/L) > palmitic acid (2444.64 mg/L) > stearic acid (2259.53 mg/L) > lauric acid (2234.35 mg/L) > acetic acid (2004.15 mg/L) > sodium octanoate (1966.5 mg/L) > sodium oleate (1904.83 mg/L) > control group (1837.33 mg/L) > myristic acid (1791.81 mg/L). Considering that oxidative stress caused by unsaturated fatty acids can have an impact on the product yield, we selected two saturated fatty acids, stearic acid and acetic acid, to further investigate the impact of fatty acids with different chain lengths on the yield of pneumocandin B_0_.

### Effect of added acetic and stearic acid on the synthesis of pneumocandin B_0_ and intracellular lipids

As a lipid-soluble fluorescent dye, Nile red can freely shuttle through the cell and bind to intracellular neutral lipids. Then, it can be directly observed through a fluorescence microscope at a specific excitation wavelength. The Nile red lipid droplet dyeing test is widely used due to its advantages of simplicity and specificity.

**Fig. 3:**
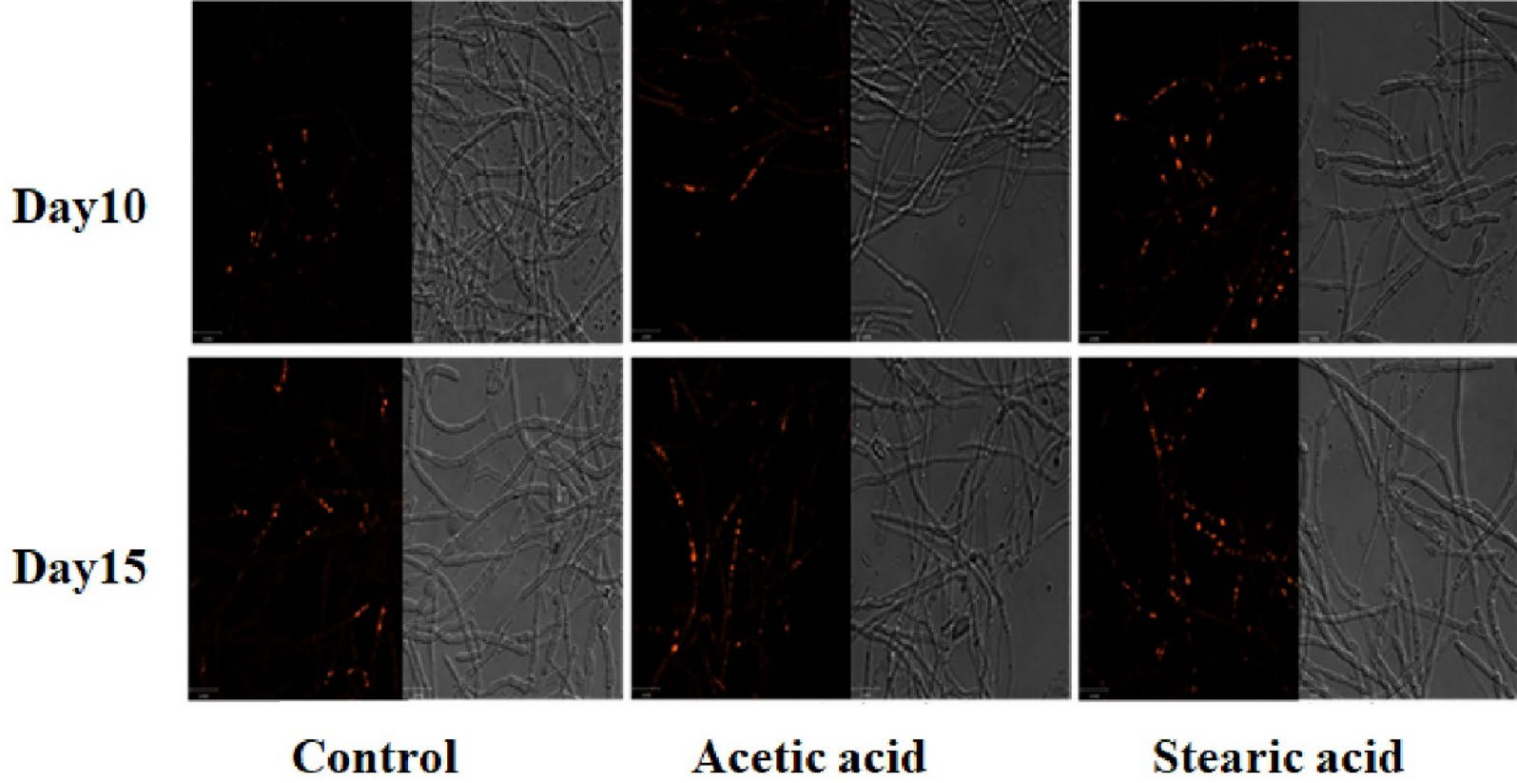
Accumulation of lipids at different timepoints during fermentation

On the 10th and 15th days of fermentation, samples were taken for Nile red lipid droplet staining and observed by fluorescence microscopy, as shown in Fig. 3. The accumulation of intracellular lipid droplets increased with time in all three groups. However, the fluorescence intensity of the stearic acid addition group was higher than that of the acetic acid addition group at the same timepoint, while the fluorescence intensity of the acetic acid addition group was higher than that of the control group. First, the experimental results showed that intracellular lipid droplets gradually increased with the fermentation time. Second, both acetic acid and stearic acid addition can promote the accumulation of intracellular lipid droplets, whereby the accumulation of lipid droplets in the stearic acid addition group was better than that in the acetic acid addition group.

Both the addition of short chain acetic acid or long chain stearic acid showed a promoting effect on the synthesis of pneumocandin B_0_ and the accumulation of intracellular lipid droplets. In the acetic acid added group, the final yield of pneumocandin B_0_ was 1981.47 mg/L, which was 7.38% higher than that of the control group. The final yield of lipids was 6836.76 mg/L, which was 6.60% higher than in the control group. In the stearic acid addition group, the final yield of pneumocandin B_0_ was 2235.57 mg/L, which was 21.15% higher than in the control group; the final yield of lipids was 7553.19 mg/L, which was 17.79% higher than in the control group, as shown in Table 1.

**Table 1:** Effect of two fatty acids on fermentation results

	pneumocandin B_0_ (mg/L)	Total lipid (mg/L)	DCW (g/L)	Proportion of extracellular pneumocandin B_0_	pH
Control	1845.26 ± 50.3	6413.73 ± 149.9	163.21 ± 8.3	9.95%	6.90 ± 0.2
Acetic acid	1981.47 ± 49.5	6836.76 ± 135.7	163.78 ± 7.3	10.73%	7.19 ± 0.2
Stearic acid	2235.57 ± 65.6	7553.19 ± 155.3	161.88 ± 5.5	10.47%	6.82 ± 0.5

**Fig. 4:**
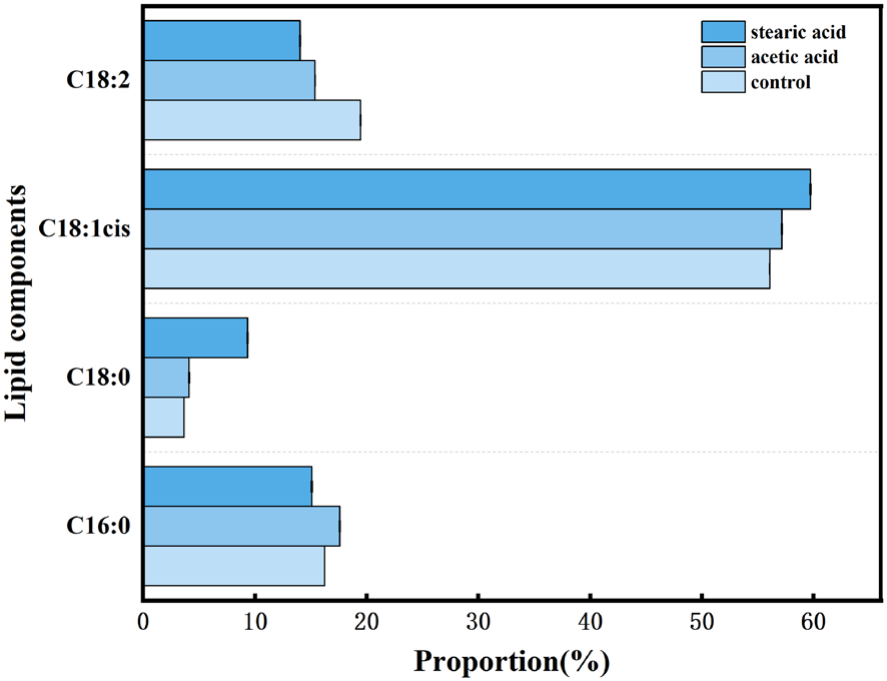
Effect of two fatty acids on lipid components

We used gas phase oil composition for testing, as shown in Fig. 4. The intracellular lipid components can be divided into four major types: C16:0, C18:0, C18:1, and C18:2. There was a slightly increase in the C16:0, C18:0, and C18:1 components in the acetic acid addition group, particularly C16:0, which increased by 11.99% relative to the control group. The greatest change in oil in the stearic acid addition group was C18:0, which increased by 154.77% relative to the control group.

### Differential gene expression in *G. lozoyensis* following the addition of acetic or stearic acid

**Fig. 5:**
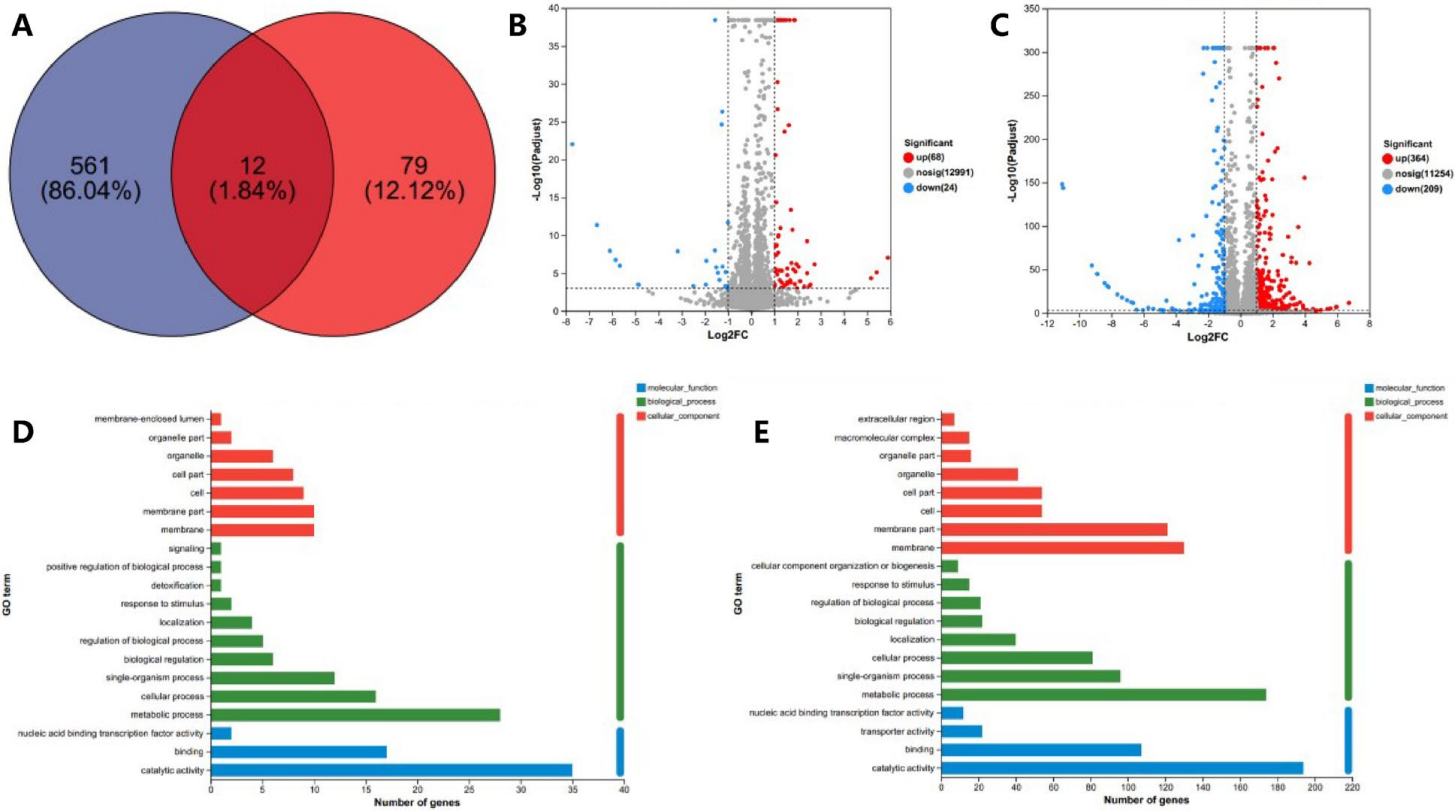
Transcriptomic profiling and DEGs annotation between the addition of acetic acid and stearic acid on *G. lozoyensis*. **A** Venn diagram illustrating the distribution of differential genes between acetic acid addition and stearic acid addition that were equally transcribed Blue: stearic acid addition, Red: acetic acid addition; **B**, **C** Scatter of differential expression gene. **B** acetic acid addition, **C** stearic acid addition; **D**, **E** Gene ontology (GO) functional enrichment analysis of DEG. The *X*-axis represents the number of DEG, and the *Y*-axis lists GO terms. **D** acetic acid addition, **E** stearic acid addition

To identify the functional genes responsible for changes in biomass and pneumocandin B_0_ following the addition of fatty acids with different carbon chain lengths, total RNA was isolated from *G. lozoyensis* and sequenced. A total of 44,147,972 reads were obtained in the control group, including 96.62% total mapped and 96.28% uniquely mapped. In the acetic acid addition group, a total of 46,171,342 reads were obtained, including 96.58% total mapped and 96.26% uniquely mapped. In the stearic acid addition group, a total of 43,666,342 reads were obtained for the stearic acid addition group, including 96.98% total mapped and 96.66% uniquely mapped. Transcriptomic profiling and differentially expressed genes (DEGs) annotation between the addition of acetic acid and stearic acid on *G. lozoyensis*, as shown in Fig. 5.

**Fig. 6:**
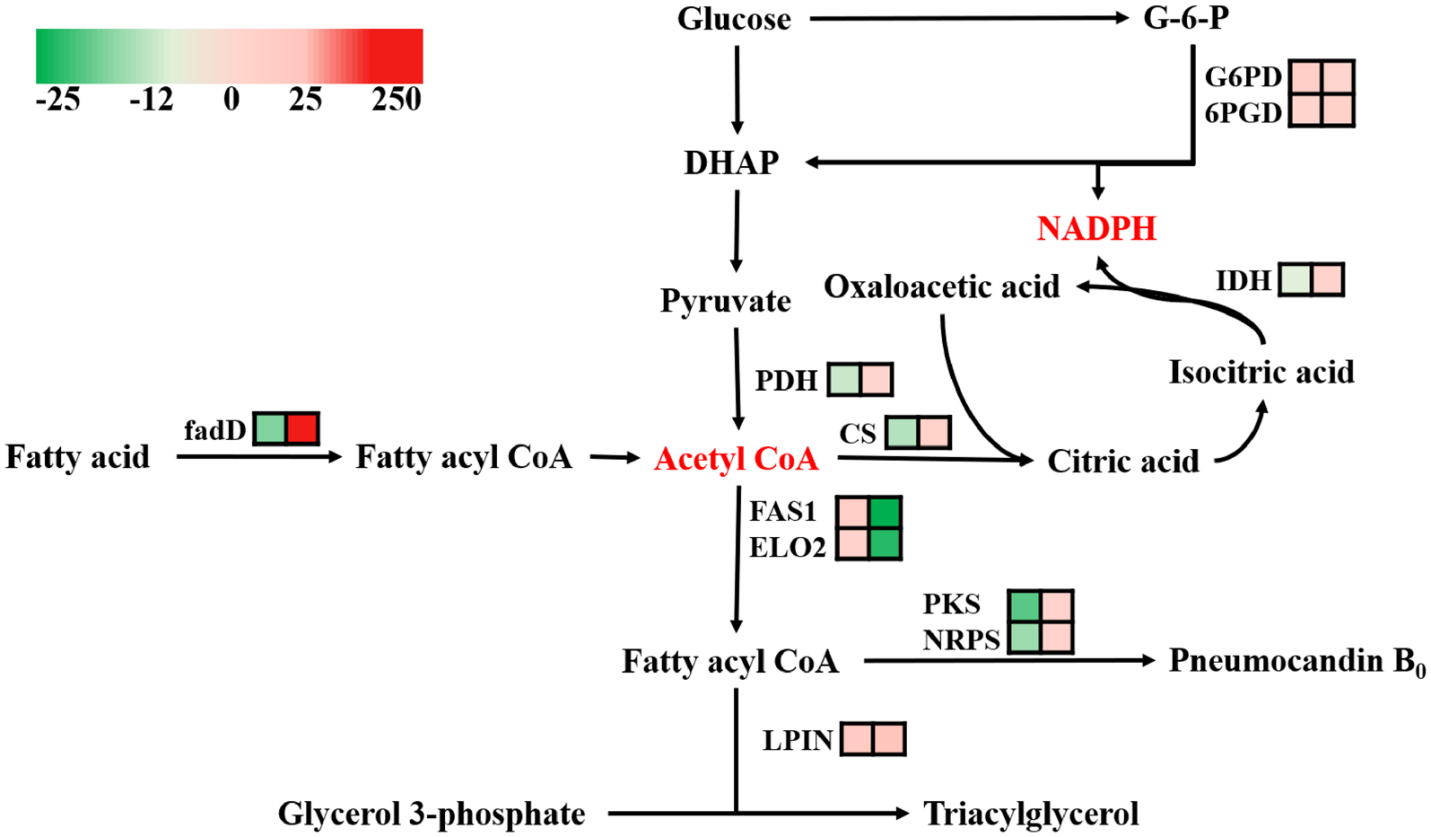
Changes in the transcript abundance of genes involved in central metabolic pathways, fatty acid biosynthesis, and the PKS–NRPS pathway. Key enzymes with logFC values are included in the map with their names written out. Red represents up- and green represents downregulation. *G6PD* glucose-6-phosphate dehydrogenase, *6PGD* 6-glucose*phosphate dehydrogenase, *IDH* isocitric dehydrogenase, *PDH* pyruvate dehydrogenase, *CS* citrate-synthase, *fadD* acyl-CoA synthetase, *FAS1* Fatty acid synthase fas1, *ELO2* fatty acid elongase 2, *PKS* polyketide synthase, *NRPS* nonribosomal peptide synthetase, *LPIN* Lipid phosphate phosphohydrolase

The transcription of genes related to pneumocandin B_0_ synthesis was downregulated, but the downregulation ratio was low. Moreover, the results showed that the final yield of pneumocandin B_0_ increased. The accumulation of intracellular pneumocandin B_0_ reached a dynamic balance. In this case, even if the transcription is upregulated and the enzyme activity is increased, the production of pneumocandin B_0_ cannot increase due to the restriction of intracellular product inhibition and the toxicity of pneumocandin B_0_. Changes in the transcript abundance of genes involved in central metabolic pathways, fatty acid biosynthesis, and the PKS–NRPS pathway, as shown in Fig. 6.

DEGs related to central carbon metabolism were mainly concentrated in the glycolytic pathway, citric acid cycle, and pentose phosphate pathway. The expression of hexokinase (HK), related to the mannitol cycle, was upregulated by 131.9% in the acetic acid group and downregulated by 26.06% in the stearic acid group. In the glycolysis pathway, except for the upregulation of pyruvate dehydrogenase (PDH) by 4.2% in the stearic acid group, the other genes were downregulated. DEGs in the citric acid cycle decreased by 2.73–8.49% under acetic acid treatment, and increased by 1.69–19.64% under stearic acid treatment. In addition, IDH increased by 3.12% in the acetic acid group and 19.64% in the stearic acid addition group. The pentose phosphate pathway is the main source of intracellular reductive power in the form of NADPH. The expression of G6PD increased by 12.3% in the acetic acid group and 3.84% in the stearic acid supplemented group. Similarly, the expression of 6PGD increased by 5.83% in the acetic acid added group and 9.65% in the stearic acid added group.

In the stearic acid supplementation group, the transcription of genes involved in the fatty acid synthesis and metabolism pathway was downregulated by 8.76% and 40.05%, while it was upregulated in the acetic acid supplementation group. The expression of fatty acid-β-oxidase (fadD) was downregulated by 2.78–29.74% in the acetic acid addition group, while it was upregulated by 354.72–415.56% in the stearic acid addition group. As mentioned in our previous work, the acetyl coenzyme A used for pneumocandin B_0_ synthesis is mainly produced by citrate lyase (ACL). In the acetic acid addition group, the ACL transcription level was decreased by 19.32%.

In the stearic acid supplementation group, the transcription level of ACL was decreased by 9.64%. Acetyl-CoA carboxylase (ACACA; FAS1) was, respectively, upregulated by 23.78% and 26.75% compared to the control group in the acetic acid supplemented group. In the stearic acid supplemented group, the transcription levels of genes involved in fatty acid synthesis and metabolism showed a downward trend, especially ACACA and FAS1, which were downregulated by 29.83% and 40.04%, respectively, while Lo2, responsible for long chain elongation, was also downregulated by 20.52%.

Fatty acylglycerol synthesis was upregulated in both experimental groups, as evidenced by the increased expression of phosphatidic acid phosphatase (LPIN; DPP1). In the acetic acid addition group, LPN increased by 17.33% and DPP1 increased by 24.46%. In the stearic acid supplemented group, LPIN was upregulated by 124.88% and DPP1 was upregulated by 32.05%. The trend was the same as that of the acetic acid addition group, and the transcription level was increased more significantly. The transcription of genes related to pneumocandin B_0_ synthesis was downregulated, but the downregulation ratio was relatively low. ABC transporters were slightly downregulated in both experimental groups (11.53%, 3.73%).

Pneumocandin B_0_ is a secondary metabolite of *G. lozoyensis* and is toxic to the producer itself. Fatty acid treatment had no effect on the proportion of extracellular pneumocandin B_0_, and there was a slight downregulation of ABC transporters, indicating that the increase in pneumocandin B_0_ was mainly due to an increase in intracellular pneumocandin B_0_, as well as an increase in the transcription levels of triacylglycerol-related genes and an increase in intracellular lipid droplets. pneumocandin B_0_ is soluble in oil, which indirectly proves that the increased lipid droplets in the cell are the “reservoir” of pneumocandin B_0_, ameliorating product feedback inhibition and toxic side effects.

## Discussion

### Addition of fatty acids to the fermentation increases pneumocandin B_0_ production and lipid droplet content

Different types of fatty acids can enhance the production of pneumocandin B_0_ and lipids during fermentation, as they can be converted to triacylglycerols in the cells and can also be utilized by polyketide synthesis enzymes. Considering the enhancing effect of soybean oil addition on the product titer in preliminary trials, we tested the addition of the major fatty acid components of soybean oil and some short-chain fatty acids. Unsaturated fatty acids resulted in higher pneumocandin B_0_ than saturated fatty acids. In the linoleic acid addition group, the final yield of pneumocandin B_0_ was increased by 46.4% compared to the control group, whereas in the oleic acid addition group, the pneumocandin B_0_ yield was only increased by 3.6% compared to the control group. In our previous study we found that when antioxidants such as vitamin C or gallic acid were added exogenously, the total intracellular antioxidant capacity was increased and the level of reactive oxygen species decreased, reducing cellular ageing and decay caused by reactive oxygen species (Song et al. 2018a, b). This phenomenon also occurs during the synthesis of polyketides. A higher content of unsaturated fatty acids in the added oil leads to higher production of polyketides. In other experiments using strains to produce antibiotics by fermentation, the addition of unsaturated fatty acids or antioxidants has been shown to enhance the final fermentation yield (Ren et al. 2017; Sun et al. 2016).

To avoid the effects of oxidative stress caused by unsaturated fatty acids, stearic acid and acetic acid were ultimately chosen as additives for further experiments. The intracellular lipid content increased with time in both the acetic and stearic acid addition groups. The lipid droplet content in the stearic acid group was superior to the acetic acid group at the same sampling time. At the end of fermentation, the yields of pneumocandin B_0_ and intracellular lipids were examined. The stearic acid group was found to have the highest pneumocandin B_0_ and lipid production, which showed a positive correlation. Examination of the major components of the oils revealed small increases in C16:0, C18:0 and C18:1 in the oil fractions of the acetic acid group, possibly due to the intracellular fatty acid synthesis pathway being facilitated by the addition of acetic acid. In the stearic acid group, the largest change in the oil was in C18:0, presumably because the exogenously added stearic acid was activated intracellularly into stearoyl-CoA, which was directly incorporated into triacylglycerols.

### Addition of fatty acids to the fermentation increases the production of NADPH in the central carbon metabolic pathway

There are three rate-limiting enzymes in glycolysis, hexokinase, fructokinase 6-phosphate (PFK) and pyruvate kinase (PK). Mannitol enters the glycolytic pathway after being generated from fructose-6-phosphate by the action of NADP^+^-mannitol 2-dehydrogenase and hexokinase. Because of the high overall level of HK downregulation, we hypothesized that the mannitol cycle was inhibited in both experimental groups. The genes encoding PFK and PK were significantly downregulated in both experimental groups, and the downregulation was more pronounced in the acetate than in the stearate group. The downregulation of glycolytic pathway genes indicates that the glycolytic pathway was inhibited and pyruvate production was reduced.

PDH converts pyruvate into acetyl-CoA, which is not only the initiator of the citric acid cycle, but also a for the synthesis of fatty acids as well as pneumocandin B_0_. The expression of PDH was downregulated in the acetic acid group and upregulated in the stearic acid group. The condensation of acetyl-CoA with oxaloacetate by citrate synthase (CS) generates citric acid and starts the citric acid cycle. Changes in the amount of the precursor substance acetyl-CoA may be associated with CS transcript levels. The expression of both CS genes was downregulated in the acetic acid group and upregulated in the stearic acid group.

In a study by Qin et al., it was found that one of the important factors influencing the synthesis of pneumocandin B_0_ is intracellular reducing power in the form of NADPH (Qin et al. 2016). ICD is responsible for the conversion of isocitric acid to oxalosuccinic acid and the concomitant generation of NADPH. This enzyme exhibited a slight increase of expression in the acetate group and a significant increase in the stearate group. The pentose phosphate pathway is the main source of intracellular reducing power in the form of NADPH. Transcriptomic data of G6PD and 6PGD suggest that the addition of both acetic acid and stearic acid promoted an active pentose phosphate pathway, providing cells with higher intracellular reducing power, which is necessary for the synthesis of pneumocandin B_0_.

### The addition of fatty acids to the fermentation promoted triacylglycerol synthesis

In *G. lozoyensis*, mannitol is fermented as a carbon source to produce pneumocandin B_0_, which undergoes a series of pathways to produce acetyl-CoA. A part of the acetyl-CoA enters the PKS–NRPS pathway to synthesize pneumocandin B_0_, and another part enters the FAS pathway to produce fatty acyl-CoA, and finally triacylglycerols. Exogenously added fatty acids are activated to fatty acyl-CoA in the first step of β-oxidation, the main route of fatty acid catabolism. Xue et al. found that overexpression of the β-oxidation pathway genes provided sufficient acetyl-CoA for the synthesis of polyketides (Xue et al. 2013). Fatty acids can be converted by fadD located in the outer mitochondrial membrane to produce high-energy, highly water-soluble fatty-acyl CoA. Upon entry into the mitochondrial matrix, the fatty acid β-oxidase system catalyzes the breakage of the fatty acyl group to produce one molecule of acetyl-CoA and one molecule of fatty acyl-CoA with two fewer carbon atoms, which continues until the fatty acid chain is shortened to acetyl-CoA. In the acetate addition group, the transcript levels of the enzymes responsible for β-oxidation were all reduced. In the stearic acid group, the transcript levels of the enzymes responsible for β-oxidation showed the same trend as the activation of fatty acids, with a certain increase in both. From the transcriptional data of the fatty acid catabolic pathway, we found that the β-oxidation pathway was inhibited in the acetic acid group, while it was very active in the stearic acid group.

Glycerol-3-phosphate is ligated with two molecules of fatty acyl-CoA in the glycerol 3-phosphate pathway to form 1,2-diacylglycerol-3-phosphate, or phosphatidic acid. The phosphatidic acid is then converted by phosphatidic acid phosphatase (LPIN; DPP1) to form 1,2-diacylglycerol, which again is reacted with acyltransferase to form triacylglycerol. Notably, the expression of phosphatidic acid phosphatase was increased in all the experimental groups. We, therefore, hypothesized that the elevated transcript levels of the enzymes in the lipid acyl synthesis pathway provide the potential for an increase in intracellular triacylglycerol synthesis. The increased lipid droplet accumulation was consistent with the transcriptomic analysis. The activated fatty acyl-CoA promoted the β-oxidation of fatty acids, providing sufficient energy for the cells, while also providing sufficient precursors for acylation, which explains the increase of lipid accumulation in the stearic acid group compared to the control and acetic acid groups.

### The addition of fatty acids to the fermentation increased pneumocandin B_0_ production

The biosynthesis of pneumocandin B_0_ begins with the assembly of myristic acid catalyzed by polyketide synthase. The polyketide synthase encoded by GLPKS4 assembles myristic acid starting from the acetyl group, loading two methyl groups to produce 10,12-dimethylmyristoyl, which is converted from free myristic acid to the thioester form by acyl AMP ligase (GLligase) and then transferred to the non-ribosomal peptide synthase GLNRPS4, which completes the assembly of six amino acids and cyclization to synthesize pneumocandin B_0_.

Although the transcript levels of the enzymes involved in the pneumocandin B_0_ synthesis pathway were all reduced, the percentage of downregulation was low. Moreover, the experimental results showed that the final yield of pneumocandin B_0_ was elevated. We found that intracellular pneumocandin B_0_ synthesis reached a dynamic equilibrium with SDS addition when the pneumocandin B_0_ yield reached 9.61 mg/g DCW (Yuan et al. 2019). In this case, even if transcript levels and enzyme activity increased, pneumocandin B_0_ production could not increase any further, as it was limited by the feedback inhibition of intracellular products and the toxic effect of pneumocandin B_0_ itself. However, there was an adequate amount of intracellular NADPH in both experimental groups, which is an important influencing factor for pneumocandin B_0_ synthesis. In addition, the increased synthesis of intracellular triacylglycerols (lipid droplets) provided storage sites for intracellular pneumocandin B_0_, and the synthesis of pneumocandin B_0_ was improved, resulting in an increased final yield at the end of the fermentation. The previous analysis showed a slight inhibition of the glycolytic pathway and the citric acid cycle, which may be responsible for the decreased transcript levels of the pneumocandin B_0_ gene cluster.

An ABC transporter protein (GLTRT) was slightly downregulated in both experimental groups, and the data also showed that the addition of exogenous fatty acids did not affect the proportion of extracellular pneumocandin B_0_, which suggests that the main reason for the increase of pneumocandin B_0_ was improved intracellular synthesis, indirectly demonstrating that the increased intracellular lipid droplets acted as a “reservoir” for pneumocandin B_0_, reducing the feedback inhibition and the toxic effects of the product.

## Conclusions

The effects of externally adding acetic acid and stearic acid on the accumulation of intracellular lipids during fermentation of *G. lozoyensis* was studied with the aim to increase pneumocandin B_0_ production. The results showed a positive correlation between the production of pneumocandin B_0_ and intracellular lipid accumulation, while the percentage of extracellular pneumocandin B_0_ did not change significantly, indicating that the increased pneumocandin B_0_ was stored intracellularly.

Through the addition of acetic or stearic acid, the triacylglycerol anabolic pathway was activated to provide sufficient synthetic precursors, promoting the synthesis of intracellular lipid droplets, which in turn provided a safe storage site for cytotoxic pneumocandin B_0_. This reduced feedback inhibition and indirectly promoted pneumocandin B_0_ synthesis, resulting in intracellular lipid droplet extraction of pneumocandin B_0_ during the fermentation.

## Data Availability

The data sets generated for this study can be found in the NCBI, Sequence Read Archive, SRP439921, https://www.ncbi.nlm.nih.gov/sra/?term=SRP439921.
